# Differential expression analysis of microRNAs and mRNAs in the mouse hippocampus of post-stroke depression (PSD) based on transcriptome sequencing

**DOI:** 10.1080/21655979.2022.2027061

**Published:** 2022-01-31

**Authors:** Fan Qinlin, Xie Qi, Chen Qiong, Xie Lexing, Shi Peixia, Hu Linlin, Diao Yupu, Yang Lijun, Yang Qingwu

**Affiliations:** a Department of Neurology, Second Affiliated Hospital of Army Medical University, Chongqing, China; b Chongqing Public Health Medical Treatment Center, Chongqing, China

**Keywords:** PSD, transcriptome sequencing, ethology, microRNA, mRNA

## Abstract

To clarify the differential expressions of microRNAs and mRNAs in a PSD model, this study employed PSD mice for model construction by injecting vasoconstrictor ET-1 (angioendothelin-1) into the medial prefrontal cortex (mPFC) of mice. The animals underwent elevated plus maze test, open field test, tail suspension test, and forced swimming test subsequently. Transcriptome sequencing was performed to analyze the differentially expressed mRNAs and microRNAs. The results showed that open arm entries and time of PSD mice were markedly decreased. Times of the entry to center for mice in the model group were apparently decreased. The climbing time of mice in the model group was greatly decreased. The behavior of PSD mice indicated a marked change, and several indicators of the behavioral tests were significantly lower than those of the control group. Transcriptome sequencing analysis demonstrated that expressions of 1 206 genes and 21 microRNAs were markedly upregulated in model group, whereas expressions of 2 113 genes and 32 microRNAs were markedly downregulated. GO analysis revealed that the differentially expressed genes were mainly involved in regulatory pathways of single-multicellular organism process, developmental process, cell periphery, plasma membrane, and neuron projection. Meanwhile, KEGG analysis results indicated that the differentially expressed genes mostly participated in signaling pathways of neuroactive ligand-receptor interaction, calcium signaling pathway, and cytokine-cytokine receptor interaction. In conclusion, differentially expressed microRNAs and mRNAs were screened, which offers a theoretical foundation for further investigation of molecular mechanisms and novel insight for the early identification, prevention, and treatment of PSD.

## 1 Introduction

Stroke represents one of the most common causes of mortality and disability globally. As a frequently encountered mental illness after stroke, PSD is responsible for impaired quality of life and rehabilitation capabilities [1,2]. PSD is defined as the depressive state suffered from a stroke. An epidemiological study on several populations has reported that PSD is a commonly seen complication of stroke. The estimated morbidity exceeds 30% within 5 years after stroke. It is reported that about 20–50% of victims are diagnosed with depression within 3 months post stroke, and most last for at least one year [1–4]. PSD affects patients’ quality of life and severely influences their rehabilitation. Despite through great efforts of research in decades, apparent progress has been achieved in pathogenesis, there is still a lack of effective treatment methods clinically. It is therefore that an in-depth investigation on the underlying PSD mechanisms and determination of its targeted effects may benefit much in clinical practice [5,6].

The hippocampus is intimately associated with human emotional and cognitive behaviors. Some studies have found that the hippocampus is not a uniform encephalic region but an intricate tissue structure, and the hippocampus formation is recognized as the basis of its relevance and functional pleiotropy [7]. Typically, the simple paradigm “cognition is controlled by the dorsal (posterior) hippocampus, while emotion is mediated by the ventral (anterior) hippocampus” attributes cognitive impairment to the dorsal hippocampus, and affective disorder depressive behavior is related to the ventral hippocampus. That is, the hippocampus is a 2-in-1 system that controls cognition and emotion in a healthy brain, and hippocampal damage may be the cause of cognitive and emotional disorders. Due to the particular structure of the hippocampus, numerous recent PSD studies have focused on the hippocampus. Previous researchers have pointed out that the hippocampus contains a great many steroid receptors GRs and MRs, which mediate the control of steroids on HPAA and behavior in a coordinated manner [8]. CS (steroids) -mediated negative feedback is achieved through the activation of GR and MR at the central level, mainly in the hippocampus. Owing to this finding, it is currently reckoned that stress activates the hypothalamus-pituitary-adrenal gland (HPA), causing excessive release of CS, which then acts on steroid receptors in the hippocampus, thereby stimulating late neuroinflammation and signal transduction. Consequently, neurogenesis disorders and hippocampus degeneration cause depression. Some current PSD research has found that acute inflammation occurs after stroke, leading to the release of glucocorticoids [8,9]. These processes reduce the transcription of neurotrophic factors, thereby reducing neurogenesis and neuroplasticity, especially in the hippocampus and frontal cortex. Additional studies have also revealed that the hippocampus volume of patients with depression is shrunk in magnetic resonance images. PSD is related to cognitive dysfunction, and this disorder may be indirectly caused by hippocampal damage.

MicroRNAs are short-stranded single-stranded ribonucleic acid molecules that bind to the 3’ untranslated region of target genes to prevent translation or promote gene degradation at the post-transcriptional level. Previous studies have shown that the expression of miR-140-5p at admission was positively correlated with the HAMD depression scale score 3 months after stroke, and the sensitivity of predicting delayed PSD is 83.3%, and specificity 72.6%[10]. A 3-month follow-up study of 251 cases of acute ischemic stroke has revealed that 45 cases (17.93%) were diagnosed with premature PSD. Three are randomly selected from the PSD group and the non-PSD group for chip sequencing. There are 25 micro-RNAs in the two groups with a multiple of difference > 2 and *P* ≤ 0.05. In the PSD group, the expression of 4 miRNAs is upregulated, and the expression of 21 miRNAs is downregulated [11].

We speculated that the regulation of transcription level was of great significance to the pathogenesis of PSD. In order to fully understand the differentially expressed mRNA and microRNA in the PSD disease model, ET-1 was injected into a PSD mouse model. Behavioral testing and hippocampal tissue sampling were performed one week after surgery, and the whole transcriptome was sequenced. Subsequently, the differential genes were screened out and specific genes of interest responsible for PSD were identified, which assisted in figuring out the pathogenesis of PSD.

## 2 Methods

### 2.1 Laboratory animals

Male C57BL/6 mice aged 6–8 weeks were purchased from Chongqing Laibite Biotechnology Co., Ltd. The animals were bred under the environment of 24 ± 2°C and 55% constant humidity. They were provided light for 12 h a day, with food and water ad libitum. Adaptive feeding to the environment was given for 2 weeks before the experiment (Figure S1A). The use of all laboratory animals and experimental protocols were approved and implemented in accordance with the statement of ethical principles developed by the World Medical Association (Declaration of Helsinki) and the Ethics Committee of Animal Testing of Xinqiao Hospital.

Establishment of PSD model: The mice were initially anesthetized with 5% halothane and maintained normal respiration with 1% halothane (a mixture of 70% N_2_O and 30% O_2_). The mouse was placed on a stereolocator (SR-9 M-HT, Narishige) and fixed, the scalp was cut open, the Bregma point was located and then adjusted to zero. The injection sites were marked at AP: 1.5 mm, ML: 0.5 mm, DP: 2.6 mm, AP: 2.0 mm, ML: 0.5 mm, and DP: 2.4 mm. The skull was treated by grinding to expose the cortex. Then 2 μg/μL ET-1 (ab158333, Abcam) injection was employed to make 1 μl in total to perform injections at the two sites, each 0.5 μL. The injections were given slowly at a rate of 0.1 μg/μL for each site [12]. After injection, the needle was kept untouched there for 5 min. After surgery when the mice woke up, they were put into the animal houses for feeding. Following 48 h, the mice were performed brain magnetic resonance diffusion imaging to determine whether the mPFC had ischemic necrosis (Figure S1B). One week after surgery, the model mice were subjected to behavioral verification to determine whether there were depressive behaviors. The control group was injected with an equal volume of PBS.

### 2.2 Behavioral assays

Elevated plus maze test: One experimental animal was placed in the central area of the elevated plus maze, the head toward the open arms, and be aware that each subsequent experimental animal should be placed in the same position. Simultaneously, the camera monitor was turned on to measure the entries of the experimental animals within 6 min, and the corresponding data were recorded. During the experiment, the operator should be at a distance of 1 meter from the maze, so that the experimental environment could be quiet and free from external disturbance (Figure S1C). SuperFst software (Xinruan, China) was employed for documentation [13].

Open field test: After putting the experimental animals into the central area of the open field, the movement of the experimental animals was recorded for 6 min, and the corresponding data were noted. During the experiment, the operator should be at a distance of 1 m from the maze, so that the experimental environment could be quiet and free from external disturbance (Figure S1D). SuperFst software (Xinruan, China) was employed for documentation [14,15].

Tail suspension test: One-third of the mouse tail was fixed with tape and hung on a bracket. The head was kept 15 cm away from the platform and photographed. The camera background showed a significant contrast with the mouse fur color, and C57 mice used a white background (Figure S1E). The test was terminated after 6 min and recorded using SuperTst software (Xinruan, China) [16].

Forced swimming test: This test is recognized as a kind of behavioral desperate experiment. By placing an animal in a confined environment, from which it struggles desperately and tries to escape but fails, thus this experiment provides an unavoidable pressure environment. After some time until the animal exhibits a typical “stationary state”, relevant parameters in the process of the motionless state of despair were observed and recorded, which were applied to evaluate the effects of depressant and antidepressant. The mice were put into a transparent plexigrine glass tank with a diameter of 10 cm and a height of 25 cm, with a water depth of 15 cm and a water temperature of 25°C. The total immobility time of the mice for 6 min was recorded, which reflected the helplessness of the animals. SuperFst software (Xinruan, China) was employed for documentation [16,17].

### 2.3 Separation of hippocampal tissue

One week after surgery, the hippocampus tissue was separated. Eight mice were tested in each experiment. They were taken out of the cage and caressed gently to be calmed down before quick decapitation at the foramen magnum. The blood on the skull surface was rinsed using 0°C – 4°C normal saline. The skin was subsequently incised to expose the skull. The skull was opened along the midline using surgical scissors, as gentle as possible to avoid damaging the brain tissue. Then the skull was clipped with hemostatic forceps from the internal to the external and gradually removed the skull bone from bottom to top. When the whole brain was exposed, the meninges and blood vessels on the brain surface were removed using ophthalmic forceps. The brain was removed from the skull floor, soaked in 0°C – 4°C normal saline for 30 s, and placed on an ice pack to isolate the hippocampus tissue of the mouse. The separated hippocampus was quickly sealed and placed in a liquid nitrogen box and transferred to a − 80°C refrigerator for freezing immediately.

### 2.4 Total RNA extraction

To each 50–100 mg of hippocampal tissue, 1 ml of Trizol (R0016, Beyotime) reagent was added and homogenated employing an electric homogenizer. The added hippocampus volume shall not exceed 10% of the Trizol reagent volume used for homogenization. To completely dissociate the nucleic acid-protein complex, the homogenized hippocampal tissue was incubated at 15°C −30°C for 5 min. To each 1 ml of hippocampal homogenate, 0.2 ml of chloroform (67–66-3, Sigma) was supplemented and vibrated the tube vigorously for 15 s. After incubation for 2–3 min at 15°C – 30°C, the mixture was centrifuged at 12,000 g for 15 min at 4°C. The upper colorless water phase was transferred to a new centrifuge tube, in which 1 ml of Trizol reagent was added during hippocampus homogenization. And 0.5 ml of isopropanol (563,935, Sigma) was added simultaneously and mixed evenly. Following incubation at 15°C – 30°C for 10 min, centrifugation was performed at 12,000 g 4°C for 10 min. The supernatant in the centrifuge tube was discarded and at least 1 ml of 75% ethanol (64–17-5, Sigma) was added to each 1 ml of hippocampal homogenate to wash the RNA precipitate. After vibration, centrifugation was performed at 7 500 g 4°C for 5 min. The upper ethanol solution in the centrifuge tube was discarded and dried the RNA precipitate in the air for 5–10 min, avoiding complete dry, otherwise the solubility of the RNA could be greatly affected. When dissolving RNA, RNase-free water (3098, Sigma) was added and repeatedly blown using a pipette several times, and incubated at 55°C – 60°C for 10 min. The RNA solution harvested was stored in a refrigerator at −80°C.

### 2.5 Transcriptome sequencing and data analysis

In transcriptome sequencing, samples of each group were mixed and tested. Transcriptome sequencing was implemented by Chongqing Biomedicine Biotechnology Co., Ltd. Original sequencing data were processed after quality control (removal of 5’ and 3’ linker sequences and filtering out < 20bp fragments) to obtain the trimmed data, which were aligned to the reference genome. Using the StringTie software, the alignment reads were mapped to known transcriptomes and transcription abundance was calculated. The Ballgown package in R software was applicable for the measurement of gene expression, and expressed as FPKM (fragments per kilobase of gene/transcript model per million mapped fragments) for unit. Where FPKM > 0.5, indicating that it was expressed in each group. Ballgown was used for quantitative analysis of expression levels and pairwise differential expression analysis. The differential fold was expressed using values of fold change (FC). FC > 1.5 and *P* < 0.05 indicated that the transcript or gene was differentially expressed between both groups. The Gene Ontology enrichment analysis method is GOseq, which is based on Wallenius non-central hyper-geometric distribution. GO enrichment takes *p* values or padj less than 0.05 as significant enrichment. KEGG (Kyoto Encyclopedia of Genes and Genomes) is the main public database about pathways. Pathway significant enrichment analysis takes KEGG Pathway as the unit and applies hypergeometric test to find pathways that are significantly enriched in candidate target genes compared with the background of the entire genome. For KEGG pathway enrichment, *p* value or padj is less than 0.05 as significant enrichment. The regulatory networks were constructed by Cytoscape V3.8 (San Diego, CA, USA).

### 2.6 qPCR detection

The extraction and quality testing of total RNA from hippocampus tissue were performed as previously described. Qualified RNA was reversely transcribed according to the 2× PCR master mix (Arraystar) instructions and cDNA was obtained. The qPCR reaction system included: 2 x Master Mix, 5 μL; upstream primer, 0.5 μL; downstream primers, 0.5 μL; template, 2 μL; ddH2O, 2 μL. The reaction conditions were 95°C, 10 min; 40 PCR cycles (95°C, 10 s; 60°C, 60 s (for fluorescence collection). The primer sequences used were as follows: FEZ1, TGCTCTGCCCTACCTAACCTT/CGCGCTTGCTCGTGTTTAAT; SCOC, CGGAGATGCTCTTGCCTATGAT/TCAATTTTCAGACCTTCCAGAGG; ULK1, CACACGCCACATAACAGACAA/GCCCCACAAGGTGAGAATAAAG; NBR1, GGGCTTGCTGTCATTCCTTCA/AAGGGAAACCGGGGAGCTAAT; and GAPDH, TGCAACCGGGAAGGAAATGAA/GCCCAATACGACCAAATCAGAG; miR-129-5p, GACAATCTTGAAGTTGTTTTCT/ATTGTCTTGAAGCTCATAAG-3; U6,CTCGCTTCGGCAGCACA/AACGCTTCACGAATTTGCGT. The primers were biosynthesized by Tsingke Biotechnology Co., Ltd. The relative expression of genes was calculated using the 2^−ΔΔCT^ method.

### 2.7 Detection of protein expression by Western blot

The hippocampus was isolated under an aseptic condition and placed in liquid nitrogen immediately. The tissues were lysed with 100 μL/mg pre-cooled RIPA lysis solution, mixed evenly in a homogenizer, lysed on ice for 15 min, vortexed for 30 s, and then lysed on ice for 15 min. The tissues were placed in a 4°C centrifuge and centrifuged at 12,000 r/min for 10 min for supernatant collection. After quantification employing Bicinchoninic acid (BCA) kit (P0012, Beyotime), PBS and loading buffer were supplemented to dilute the final protein concentration to 2.5 μg/μL. The protein samples were denatured for incubation at 100°C for 5 min, and frozen at −80°C for later use. Electrophoresis was subsequently performed using 10% SDS-PAGE gel. Concentrated gel electrophoresis was run at 80 V constant voltage for 30 min, and separate gel at 120 V constant voltage for 90 min. The protein was wet transferred to a PVDF membrane under 70 V constant pressure for 90 min. Following protein transfer, a 5% skimmed milk powder TBST solution was supplemented for blockage at room temperature for 1 h and primary antibodies (anti-FEZ1, 1:5 000, Abcam, USA, anti-SCOC, 1:500, Abcam, USA; anti-ULK1, 1:2 000, Abcam USA; anti-NBR1, 1:1 000, Abcam, USA; and anti-GAPDH, 1:3 000, Bioss, China) were added for incubation in a shaker at 4°C and vibrated gently overnight. On the second day, the membrane was washed 3 times with TBST, each time for 10 min; After secondary antibody was incubated for 2 h, it was repeatedly incubated in an ultra-sensitive luminescence color developing solution for 1 min and then developed in a gel imaging system. ImageJ (Rawak Software, Germany) software was employed for gray value analysis of images.

### 2.8 Statistical analysis

Statistical software Graphpad Prism 8.0 (Graphpad, USA) was used to analyze the data. The results were statistically analyzed by One-way ANOVA followed by Tukey’s post test. Measurement data were expressed by mean ± SEM. *P* < 0.05 was considered statistically significant.

## 3 Results

PSD is the most common mental problem after stroke, which seriously affects the quality of life and rehabilitation ability of patients. We speculated that the regulation of transcription level was of great significance to the pathogenesis of PSD. In order to clarify the differential expression of microRNAs and mRNAs in the hippocampus of the PSD model, this study prepared a PSD mouse model by injection of ET-1. one week after the operation, behavioral testing were performed and hippocampal tissue were collected for transcriptome sequencing.

### 3.1 Establishment of PSD model and behavioral assays

**Figure 1.**
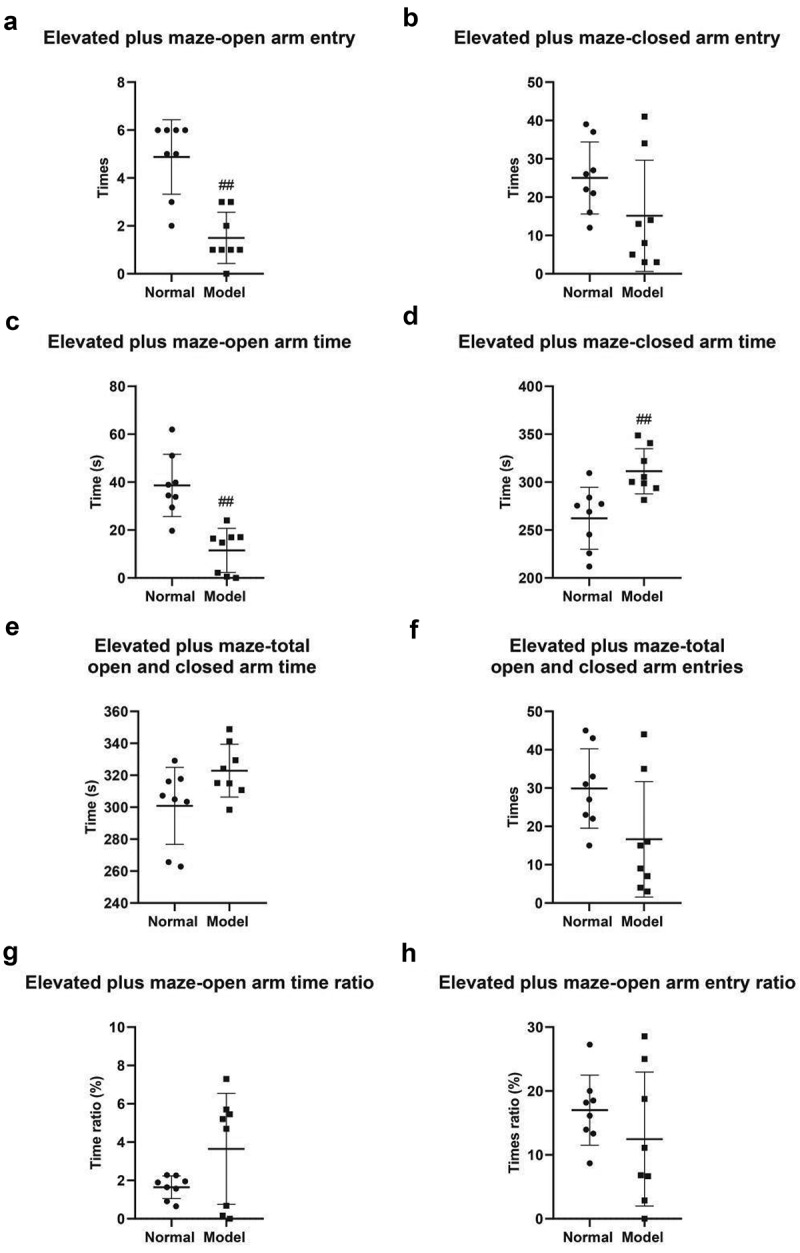
Elevated plus maze test. A, Times of open arm entry. B, Times of closed arm entry. C, Open arm time. D, Closed arm time. E, Duration of open and closed arm time. F, Total numbers of open and closed arm entries. G, Time ratio of open arm entry. H, Time ratio of closed arm entry. #, *P* < 0.05; ##, *P* < 0.01.

**Figure 2.**
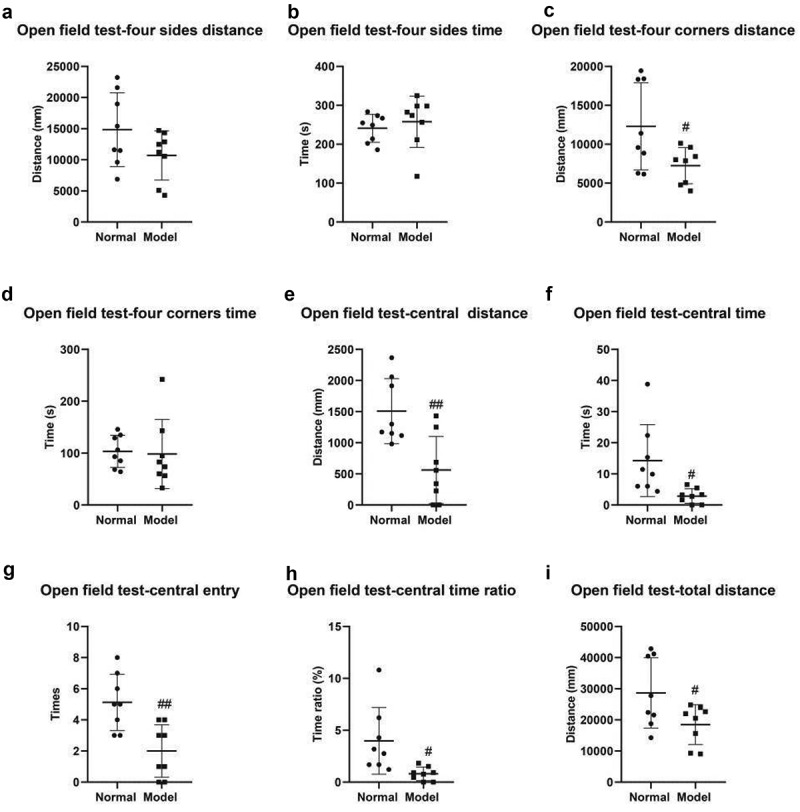
Open field test. A, Track length of mice in four edges of the open field. B, Track time of mice in four edges. C, Track length of mice in four corners. D, Track time of mice in four corners. E, Track length of mice in the center. F, Time of mice entry to center. G, Times of the mice entry to center of the open field. H, Time ratio of mice entry to center of the open field. I, Total track length of mice. #, *P* < 0.05; ##, *P* < 0.01.

**Figure 3.**
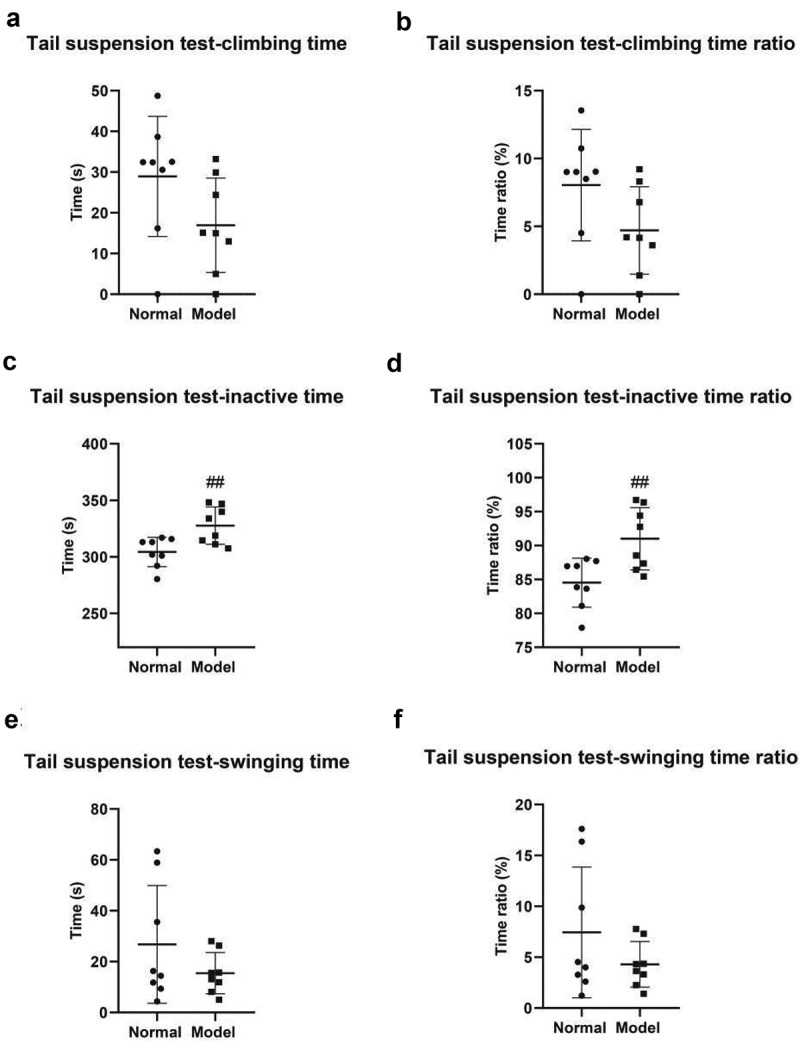
Tail suspension test. A, Climbing duration of mice in both groups. B, Climbing time ratio. C, Inactive time. D, Inactive time ratio. E, Swing time. F, Swing time ratio. #, *P* < 0.05; ##, *P* < 0.01.

This study established a PSD mouse model by injecting vasoconstrictor ET-1 (angioendothelin-1) into the medial prefrontal cortex (mPFC) of mice. Meanwhile, behavioral changes of the mouse model were observed and measured using elevated plus maze tests, open field tests, tail suspension tests, and forced swimming tests. The results of elevated plus maze tests indicated that the times of open arm entry of mice were 4.88 ± 0.55 in the normal group and 1.50 ± 0.38 in the model group (*P* < 0.01) (Figure 1a); The times of closed arm entry of mice were 25.00 ± 3.33 in the normal group and 15.13 ± 5.14 in the model group (Figure 1b); The open arm time of mice was 38.65 ± 4.60 s in the normal group and 11.49 ± 3.25 s in the model group (*P* < 0.01) (Figure 1c); The closed arm time of mice was 262.20 ± 11.42 s in the normal group and 311.40 ± 8.34 s in the model group (*P* < 0.01) (Figure 1d); The total open arm time of mice was 300.90 ± 8.52 s in the normal group and 322.90 ± 5.86 s in the model group (Figure 1e); The total closed arm time of mice was 29.88 ± 3.67 s in the normal group and 16.63 ± 5.33 s in the model group (Figure 1f); The time ratio of open arm entry was 1.65% ± 0.21% in the normal group and 3.65% ± 1.03% in the model group (Figure 1g); The open arm entry ratio of mice was 17.01% ± 1.94% in the normal group and 12.47% ± 3.71% in the model group (Figure 1h);

The results of open field tests indicated that the track length of four edges was 14,850 ± 2 093 mm in the normal group and 10,701 ± 1 402 mm in the model group (Figure 2a); The track time of four edges was 241.2 ± 12.73 s in the normal group and 257.9 ± 23.31 s in the model group (Figure 2b); The track length of mice in four corners was 12,308 ± 1 798 mm in the normal group and 7 237 ± 819.9 mm in the model group (P < 0.05) (Figure 2c); The track time of mice in four corners was 103.4 ± 10.93 s in the normal group and 98.26 ± 23.54 s in the model group (Figure 2d); The track length of mice in the center was 1 506 ± 185 mm in the normal group and 561.6 ± 190.7 mm in the model group (*P* < 0.01) (Figure 2e); The track time of mice in central entry was 14.28 ± 4.08 s in the normal group and 2.87 ± 0.83 s in the model group (*P* < 0.05) (Figure 2f); The times of entry to center were 5.13 ± 0.64 in the normal group and 2.00 ± 0.60 in the model group (*P* < 0.01) (Figure 2g); The time ratio of entry to center was 3.98% ± 1.13% in the normal group and 0.80% ± 0.23% in the model group (*P* < 0.05) (Figure 2h); The total track length of mice was were 28,664 ± 3 992 in the normal group and 18,500 ± 2 255 in the model group (*P* < 0.05) (Figure 2i);

The tail suspension test revealed that the climbing time of mice was 28.94 ± 5.22 s in the normal group and 16.93 ± 4.10 s in the model group (Figure 3a); The proportion of climbing time was 8.04 ± 1.45% in the normal group and 4.70% ± 1.14% in the model group (*P* < 0.05) (Figure 3b); The inactive time was 304.30 ± 4.60 s in the normal group and 327.60 ± 5.84 s in the model group (*P* < 0.01) (Figure 3c); The inactive time ratio was 84.53 ± 1.28% in the normal group and 91.00 ± 1.63% in the model group (*P* < 0.01) (Figure 3d); The swing time was 26.76 ± 8.17 s in the normal group and 15.46 ± 2.85 s in the model group (Figure 3e); The proportion of swing time was 7.44 ± 2.27% in the normal group and 4.30 ± 0.79% in the model group (Figure 3f).

**Figure 4.**
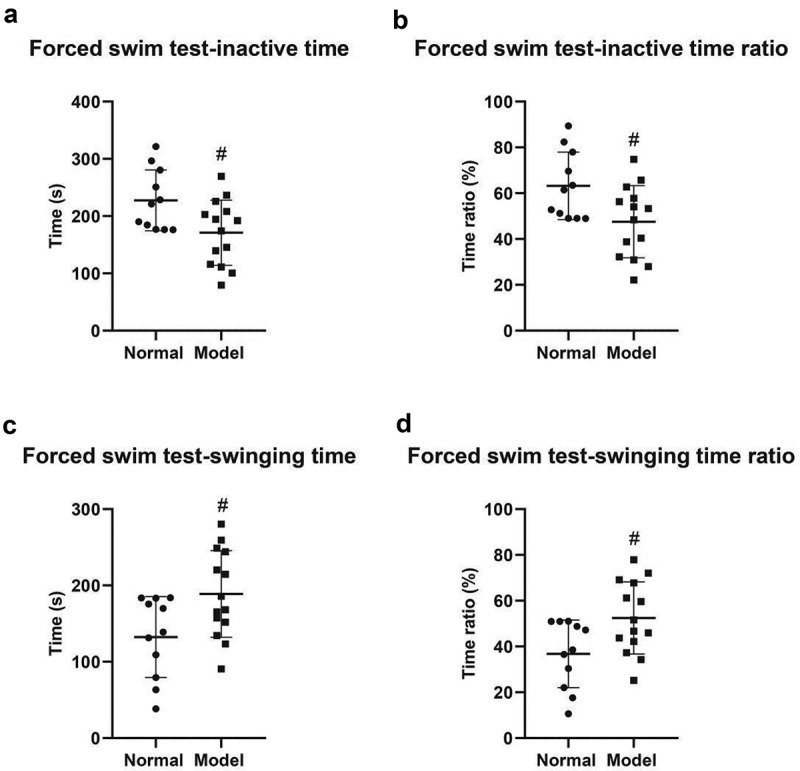
Forced swimming test. A, Inactive time of mice in both groups. B, Inactive time ratio. E, Swimming time. D, Swimming time ratio. #, *P* < 0.05; ##, *P* < 0.01.

Finally, in forced swimming tests, the inactive time of the two groups was 132.5 ± 15.99 s in the normal group and 188.9 ± 15.18 s in the model group (*P* < 0.05) (Figure 4a); And the inactive time ratio was 36.79 ± 4.44% in the normal group and 52.47 ± 4.22% in the model group (*P* < 0.05) (Figure 4b); The swing duration was 227.6 ± 15.99 s in the normal group and 171.1 ± 15.18 s in the model group (*P* < 0.05) (Figure 4c); The proportion of swing time was 63.21 ± 4.44% in the normal group and 47.53 ± 4.22% in the model group (*P* < 0.05) (Figure 4d). The previously described results revealed that compared with the control mice, the behavior of PSD mice indicated a marked change and several indicators of the behavioral tests were markedly lower than those of the control group. It indicated that the PSD mouse model was successfully prepared in this study.

### 3.2 Transcriptome sequencing to screen differentially expressed mRNA and microRNA in PSD mice

**Figure 5.**
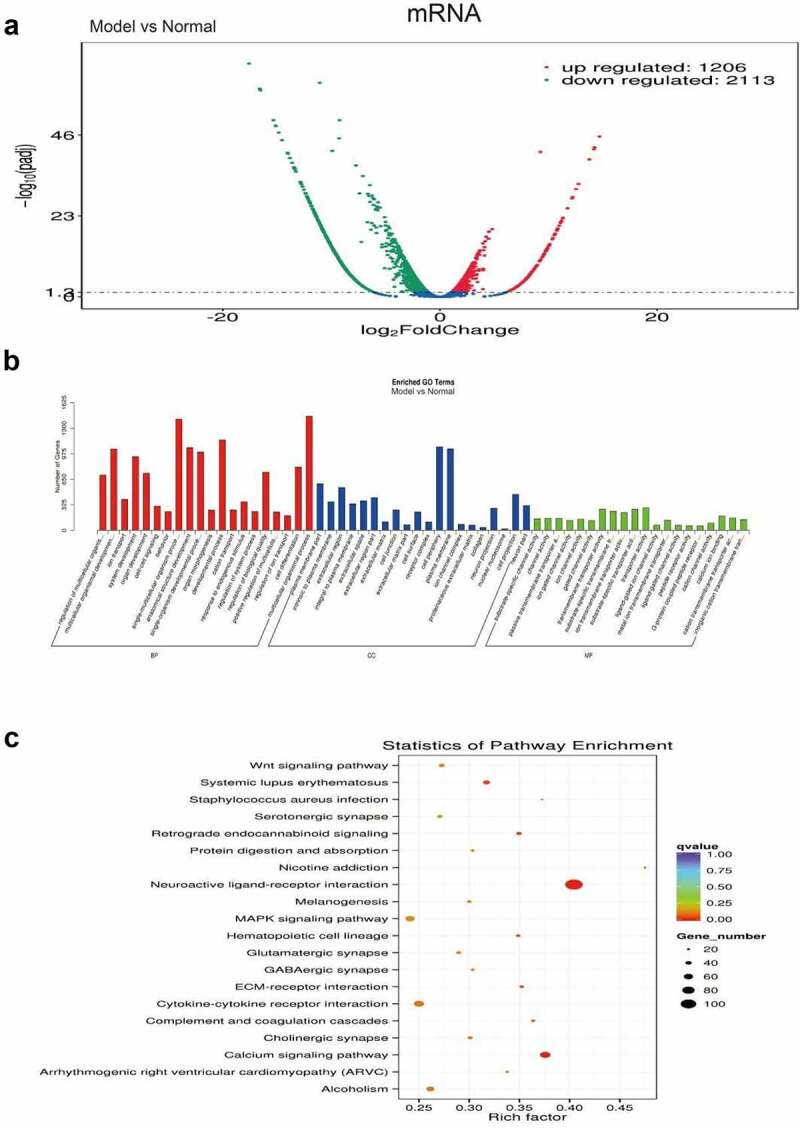
Transcriptome sequencing screening the differentially expressed mRNA in post-stroke depression mice. A, Differential gene volcano map. A total of 1206 genes were significantly upregulated and 2 113 genes were significantly downregulated. B. GO analysis. C, KEGG analysis of major signaling pathways involved in differentially expressed genes.

The previously performed tests were phenotypic research of the behavioral changes of PSD mice. To further reveal the effect of PSD on the molecular level of mice, we used transcriptome sequencing to detect the differential expressions of PSD mice at a transcriptome level. The results showed that compared with the control group, the expression of 1 206 genes in the model group was significantly upregulated, and the expression of 2 113 genes was significantly downregulated (Figure 5a). GO analysis showed that the differentially expressed genes were mainly involved in single-multicellular organism process, developmental process, cell periphery, plasma membrane, and neuron projection, and other regulatory pathways (Figure 5b). KEGG analysis results showed that differentially expressed genes were mainly involved in neuroactive ligand-receptor interaction, calcium signaling pathway, and cytokine-cytokine receptor interaction, and other signaling pathways (Figure 5c).

**Figure 6.**
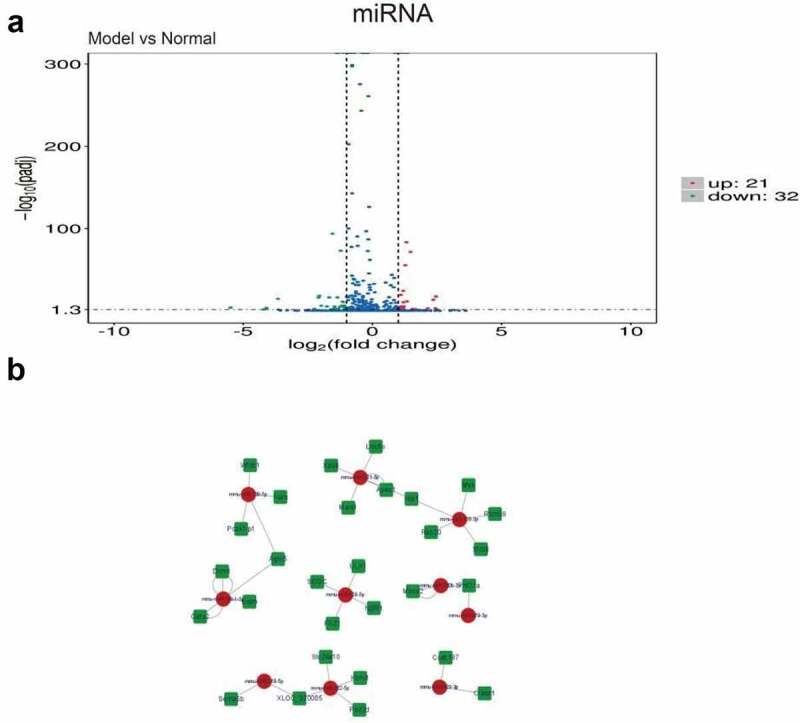
Transcriptome sequencing screened differentially expressed microRNAs and targeted relationships between differentially expressed microRNAs and significantly different mRNAs in post-stroke depression mice. A, Differentially expressed microRNA volcano map. B, Targeting relationship between significantly differentially expressed microRNAs and significantly different mRNAs.

In addition, through transcriptome sequencing, it was found that the expression of 21 microRNAs in the model group was significantly upregulated, and the expression of 32 microRNAs was significantly downregulated (Figure 6a). We conducted a joint targeted analysis of differentially expressed microRNAs and mRNA and tried to dig out the regulatory network. The results were shown in Figure 6b, showing the targeting relationship of some significantly and differentially expressed microRNAs and significantly different mRNAs. From this, we speculated that PSD mice altered greatly in the transcription level compared with the control.

### 3.3 The expression of multiple autophagy indicators increases significantly in the PSD mouse model

**Figure 7.**
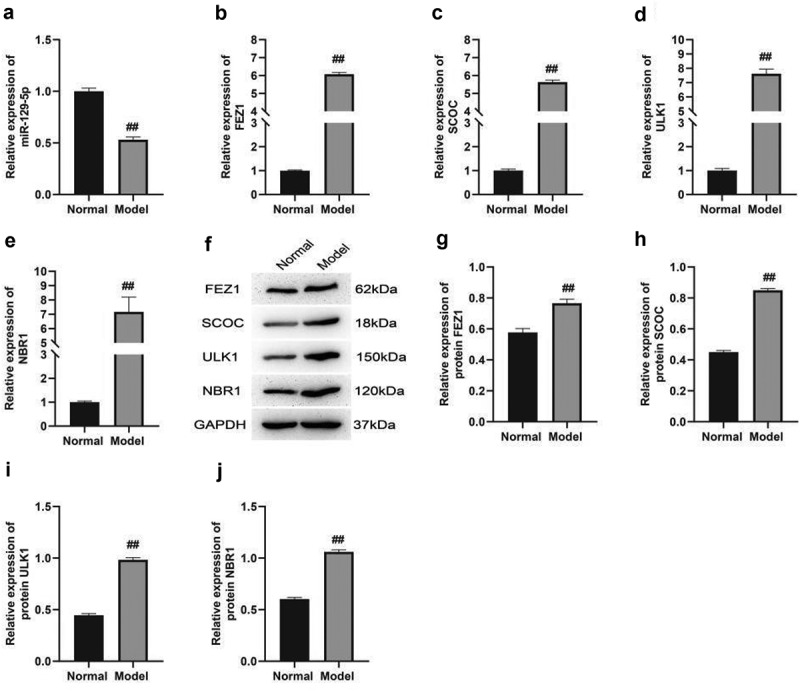
qPCR and Western blot verification of the sequencing results. A, qPCR detection of miR-129-5p expression. B-E, qPCR detection of the expressions of FEZ1, NBR1, ULK1 and SCOC. F-J, Western blot detection of the protein expression of FEZ1, NBR1, ULK1 and SCOC. #, *P* < 0.05; ##, *P* < 0.01.

It was observed from Figure 6 that the four genes FEZ1, NBR1, ULK1, and SCOC around miR-129-5p, were closely related to nervous system autophagy, which have also been reported in the literature and increased autophagy has also been reported in the hippocampus of PSD mice. To further clarify this point, we

detected the expressions of miR-129-5p, FEZ1, NBR1, ULK1, and SCOC in the hippocampus of the model group and the control group. The qPCR results showed that in the hippocampus of PSD mice, the expression of miR-129-5p was significantly reduced (Figure 7a), and the expressions of FEZ1, NBR1, ULK1, and SCOC were significantly increased (Figure 7b-7e). The protein expression levels of FEZ1, NBR1, ULK1, and SCOC were detected by Western blot, and the results were consistent with qPCR results. In the hippocampus tissue of PSD mice, the expressions of FEZ1, NBR1, ULK1, and SCOC protein increased significantly (figure 7f-7j). It was verified by qPCR and Western blot experiments which were consistent with the results obtained by sequencing. In addition, miR-129-5p and autophagy-related genes FEZ1, NBR1, ULK1, and SCOC can be used as the direction of our subsequent research to further investigate its functions and molecular regulatory mechanisms.

## 4 Discussion

Depression is the most common neuropsychiatric complication after stroke. Nearly 33% of stroke survivors suffer from PSD [18,19]. Its core symptoms are depression, apathy, fatigue, insomnia, low self-evaluation, and even suicide. Early identification, prevention, and treatment of PSD are critical to the recovery and prognosis of stroke survivors [20–22]. In order to clarify the behavioral and transcriptional changes of PSD, the vasoconstrictor ET-1 was injected into the mPFC to establish a PSD mouse model. Behavioral tests elevated plus maze, open field, tail suspension, and forced swimming were performed. Differentially expressed mRNA and microRNA in model mice were analyzed by transcriptome sequencing and verified the sequencing results. This study found that the PSD behavioral test indicators of mice were substantially lower than those of the control group, and the expression levels of 1 206 genes were significantly upregulated, and the expression levels of 2 113 genes were markedly downregulated; the expression levels of 21 microRNAs were significantly upregulated, and the expression levels of 32 microRNAs were significantly downregulated. In addition, this study indicated that miR-129-5p and autophagy-related genes FEZ1, NBR1, ULK1, and SCOC could be used as the direction of our future research to explore the functions and molecular regulation mechanisms.

As human brain tissue is difficult to be collected, it is easier to apply PSD animal models to study related brain regions. A model combining middle cerebral artery occlusion (MCAO) with chronic mild stress evokes stress- and stroke-induced behavioral phenotypes that can be treated with antidepressants [23–25]. However, MCAO models are usually associated with large and variable lesions that affect large areas of the cortex and striatum, and can therefore be confused by obvious movement disorders that interfere with the assessment of depression and anxiety [12]. The model we currently applied has nothing to do with sports injuries and persists over time. Microinjection of ET-1 into the left mPFC of male C57/BL6 mice can produce unilateral and reproducible focal ischemic injury, leading to strong anxiety and depression phenotypes without sensorimotor impairment [12]. These findings have indicated that mPFC’s ET-1 damage may help detect local brain activity changes that lead to the PSD phenotype, and provide help for us to further study the pathogenesis of PSD.

In the study of brain areas related to PSD, the hippocampus is the tissue that attracted more concerns [26,27]. Nerve fibers from the hippocampus can project to the prefrontal cortex, amygdala, and other brain areas related to emotions. The hippocampus is the high regulatory center of the stress response, and it is also a sensitive brain area that is easily affected by the stress response. Meanwhile, it is closely related to the occurrence of depression. Studies have shown that metabonomics technology can be applied to analyze the depression-related metabolites in the hippocampus of depression rats induced by chronic unpredictable mild stimuli, suggesting that the onset of depression links to energy metabolism, amino acid metabolism, and lipid metabolism [26,27]. Additionally, the enrichment of circRNAs and miRNAs specifically expressed in rats in the depression group and the control group is also related to energy metabolism and lipid metabolism. Furthermore, another study has detected the expressions of two autophagy-related proteins, LC3 and Beclin1, in the hippocampus of depression rats with chronic unpredictable mild stimulation, and found that they were significantly increased in the hippocampus of depression rats [28,29]. Our research also indicated that the expression of 21 microRNAs was markedly upregulated, and the expression of 32 microRNAs was significantly downregulated. Moreover, the expressions of FEZ1, NBR1, ULK1, SCOC, and other proteins related to autophagy increased substantially.

In recent years, a large number of studies have shown that microRNAs are involved in the pathophysiological process of many diseases, especially in cerebral infarction, cancer, neurodegenerative diseases, and epilepsy. There have been some studies on the relationship between miRNAs and stroke and depression in the past [30,31]. A study of 251 patients with acute cerebral infarction includes early-onset PSD, late-onset PSD, and non-depressive groups according to whether PSD occurred 2 weeks and 3 months after the onset [32]. Using gene chip technology to identify the differential expression profile of serum miRNAs, the differentially expressed miR-140-5p in patients with late-onset PSD was significantly upregulated. miR140-5p is an independent risk factor for late-onset depression, and the expression of miR-140-5p at admission was positively correlated with the HAMD score 3 months after stroke. In a high-throughput sequencing study of peripheral blood miRNAs involving 20 cases of acute ischemic stroke and matched healthy individuals, it was found that miR-125a-5p, miR-125b-5p, and miR-143-3p were combined to diagnose acute ischemic stroke. The area under the ROC curve of ischemic stroke patients and healthy controls is 0.90 (sensitivity: 85.6%; specificity: 76.3%), which is superior to conventional multi-modal brain CT (sensitivity: 72.5%). MicroRNAs can regulate one or more target genes, and the same target gene may be regulated by multiple microRNAs. If the target gene of a microRNA is a post-stroke depression-related gene, the expression of this microRNA may regulate the occurrence and development of PSD. This study demonstrated that in the PSD mouse model, the expression of 1 206 genes was significantly upregulated, whereas the expression of 2 113 genes was significantly downregulated; the expression of 21 microRNAs was significantly upregulated, but the expression of 32 microRNAs was significantly downregulated. GO analysis showed that differentially expressed genes were mainly involved in single-multicellular organism process, developmental process, cell periphery, plasma membrane, and neuron projection, and other regulatory pathways. KEGG analysis showed that differentially expressed genes were mostly involved in neuroactive ligand-receptor interaction, calcium signaling pathway, and cytokine-cytokine receptor interaction, and other signaling pathways. Additionally, this study found that miR-129-5p and autophagy-related genes FEZ1, NBR1, ULK1, and SCOC could be used as the direction of our next research to further study its functions and molecular regulatory mechanisms. The limitation of this study is the failure to perform further functional verification of differentially expressed microRNAs and mRNA. After overexpression or knockout of candidate genes at the animal and cellular levels, the phenotypic changes of the animal model or cell model and the changes of the marker protein need to be detected.

## 5 Conclusions

The behavior of PSD mice indicated a marked change, and several indicators of the behavioral tests were markedly lower than those of the control group. The behavior of depressed mice after a stroke had changed significantly, and many behavioral test indicators were significantly lower than the control group. A total of 21 microRNAs expression levels were substantially upregulated, and 32 microRNAs expression levels were markedly downregulated. miR-129-5p and autophagy-related genes FEZ1, NBR1, ULK1, and SCOC could be used as targets for further research.

## Highlights

The present study successfully developed a mouse model of PSD.

Differentially expressed microRNAs were screened in PSD mouse model.

Differentially expressed mRNAs were screened in PSD mouse model.

## References

[1] Medeiros GC, Roy D, Kontos N, et al. Post-stroke depression: a 2020 updated review. Gen Hosp Psychiatry. 2020;66:70–80. 32717644 10.1016/j.genhosppsych.2020.06.011

[2] Starkstein SE, Hayhow BD. Treatment of Post-Stroke Depression. Curr Treat Options Neurol. 2019;21(7):31. 31236751 10.1007/s11940-019-0570-5

[3] Sarkar A, Sarmah D, Datta A, et al. Post-stroke depression: chaos to exposition. Brain Res Bull. 2021;168:74–88. 33359639 10.1016/j.brainresbull.2020.12.012

[4] Zhang E, Liao P. Brain-derived neurotrophic factor and post-stroke depression. J Neurosci Res. 2020;98(3):537–548. 31385340 10.1002/jnr.24510

[5] Cai W, Mueller C, Li YJ, et al. Post stroke depression and risk of stroke recurrence and mortality: a systematic review and meta-analysis. Ageing Res Rev. 2019;50:102–109. 30711712 10.1016/j.arr.2019.01.013

[6] Ilut S, Stan A, Blesneag A, et al. Factors that influence the severity of post-stroke depression. J Med Life. 2017;10(3):167–171. 29075345 PMC5652262

[7] Weems CF, Russell JD. Variation in the developing brain and the role of pediatric posttraumatic stress on structural and functional networks. Biol Psychiatry Cogn Neurosci Neuroimaging. 2020;5(1):7–9. 31918892 10.1016/j.bpsc.2019.10.003 PMC7393675

[8] Hu J, Zhou W, Zhou Z, et al. miR-22 and cerebral microbleeds in brainstem and deep area are associated with depression one month after ischemic stroke. Braz J Med Biol Res. 2020;53(5):e9162. 32348425 10.1590/1414-431X20209162 PMC7197650

[9] Hu J, Zhou W, Zhou Z, et al. Elevated neutrophil-to-lymphocyte and platelet-to-lymphocyte ratios predict post-stroke depression with acute ischemic stroke. Exp Ther Med. 2020;19(4):2497–2504. 32256727 10.3892/etm.2020.8514 PMC7086204

[10] Liang HB, He JR, Tu XQ, et al. MicroRNA-140-5p: a novel circulating biomarker for early warning of late-onset post-stroke depression. J Psychiatr Res. 2019;115:129–141. 31129437 10.1016/j.jpsychires.2019.05.018

[11] Zhang Y, Cheng L, Chen Y, et al. Clinical predictor and circulating microRNA profile expression in patients with early onset post-stroke depression. J Affect Disord. 2016;193:51–58. 26766035 10.1016/j.jad.2015.12.061

[12] Vahid-Ansari F, Lagace DC, Albert PR. Persistent post-stroke depression in mice following unilateral medial prefrontal cortical stroke. Transl Psychiatry. 2016;6(8):e863. 27483381 10.1038/tp.2016.124 PMC5022078

[13] Shoji H, Miyakawa T. Effects of test experience, closed-arm wall color, and illumination level on behavior and plasma corticosterone response in an elevated plus maze in male C57BL/6J mice: a challenge against conventional interpretation of the test. Mol Brain. 2021;14(1):34. 33588907 10.1186/s13041-020-00721-2 PMC7885464

[14] Chen T, Shou L, Guo X, et al. Magnolol attenuates the locomotor impairment, cognitive deficit, and neuroinflammation in Alzheimer’s disease mice with brain insulin resistance via up-regulating miR-200c. Bioengineered. 2022;13(1):531–543. 34968163 10.1080/21655979.2021.2009975 PMC8805894

[15] Tang M, Chen M, Li Q. Paeoniflorin ameliorates chronic stress-induced depression-like behavior in mice model by affecting ERK1/2 pathway. Bioengineered. 2021;12(2):11329–11341. 34872456 10.1080/21655979.2021.2003676 PMC8810059

[16] Si L, Wang Y, Liu M, et al. Expression and role of microRNA-212/nuclear factor I-A in depressive mice. Bioengineered. 2021;12(2):11520–11532. 34889698 10.1080/21655979.2021.2009964 PMC8810195

[17] Shannon P, Markiel A, Ozier O, et al. Cytoscape: a software environment for integrated models of biomolecular interaction networks. Genome Res. 2003;13:2498–2504. 14597658 10.1101/gr.1239303 PMC403769

[18] Ahmed ZM, Khalil MF, Kohail AM, et al. The prevalence and predictors of post-stroke depression and anxiety during COVID-19 pandemic. J Stroke Cerebrovasc Dis. 2020;29(12):105315. 32958396 10.1016/j.jstrokecerebrovasdis.2020.105315 PMC7834239

[19] Ibrahimagic OC, Smajlovic D, Kunic S, et al. Post-stroke depression. Mater Sociomed. 2019;31(1):31–34. 31213952 10.5455/msm.2019.31.31-34 PMC6511370

[20] Ghaffari A, Akbarfahimi M, Rostami HR. Discriminative factors for post-stroke depression. Asian J Psychiatr. 2020;48:101863. 31901586 10.1016/j.ajp.2019.101863

[21] Trofimova SA, Balunov OA, Dubinina EE. [Oxidative stress and post-stroke depression]. Zh Nevrol Psikhiatr Im S S Korsakova. 2020;120:44–49. 10.17116/jnevro202012007144 32790975

[22] Schottke H, Gerke L, Dusing R, et al. Post-stroke depression and functional impairments - A 3-year prospective study. Compr Psychiatry. 2020;99:152171. 32179262 10.1016/j.comppsych.2020.152171

[23] Lin S, Luan X, He W, et al. Post-stroke depression and estimated glomerular filtration rate: a prospective stroke cohort. Neuropsychiatr Dis Treat. 2020;16:201–208. 32021214 10.2147/NDT.S225905 PMC6982452

[24] Wu D, Zhang G, Zhao C, et al. Interleukin-18 from neurons and microglia mediates depressive behaviors in mice with post-stroke depression. Brain Behav Immun. 2020;88:411–420. 32272223 10.1016/j.bbi.2020.04.004

[25] Castilla-Guerra L, Fernandez MM, Esparrago-Llorca G, et al. Pharmacological management of post-stroke depression. Expert Rev Neurother. 2020;20(2):157–166. 31860359 10.1080/14737175.2020.1707666

[26] Cuijpers P, Quero S, Dowrick C, et al. Psychological treatment of depression in primary care: recent developments. Curr Psychiatry Rep. 2019;21(12):129. 31760505 10.1007/s11920-019-1117-x PMC6875158

[27] Alexopoulos GS. Depression in the elderly. Lancet. 2005;365(9475):1961–1970. 15936426 10.1016/S0140-6736(05)66665-2

[28] Yang LN, Pu JC, Liu LX, et al. Integrated metabolomics and proteomics analysis revealed second messenger system disturbance in hippocampus of chronic social defeat stress rat. Front Neurosci. 2019;13:247. 30983951 10.3389/fnins.2019.00247 PMC6448023

[29] Li P, Hao XC, Luo J, et al. Propofol mitigates learning and memory impairment after electroconvulsive shock in depressed rats by inhibiting autophagy in the hippocampus. Med Sci Monit. 2016;22:1702–1708. 27203836 10.12659/MSM.897765 PMC4917309

[30] Fang M, Zhong L, Jin X, et al. Effect of inflammation on the process of stroke rehabilitation and poststroke depression. Front Psychiatry. 2019;10:184. 31031649 10.3389/fpsyt.2019.00184 PMC6470379

[31] Yan H, Fang M, Liu XY. Role of microRNAs in stroke and poststroke depression. ScientificWorldJournal. 2013;2013:459692. 24363618 10.1155/2013/459692 PMC3865697

[32] Liang HB, He JR, Tu XQ, et al. MicroRNA-140-5p: a novel circulating biomarker for early warning of late-onset post-stroke depression. J Psychiatr Res. 2019;115:129–141. 31129437 10.1016/j.jpsychires.2019.05.018

